# Spatial distribution and density of fibroblasts determine angiogenic response of endothelial cells

**DOI:** 10.21203/rs.3.rs-9705878/v1

**Published:** 2026-06-08

**Authors:** Pu Zhang, Melur K. Ramasubramanian, Li-Heng Cai

**Affiliations:** 1 Soft Biomatter Laboratory, Department of Material Science and Engineering, University of Virginia, Charlottesville, VA 22904, USA; 2 Department of Mechanical and Aerospace Engineering, University of Virginia, Charlottesville, VA 22904, USA; 3 Department of Chemical Engineering, University of Virginia, Charlottesville, VA 22904, USA; 4 Department of Biomedical Engineering, University of Virginia, Charlottesville, VA 22904, USA; 5 Department of Chemistry, University of Virginia, Charlottesville, VA 22904, USA

**Keywords:** Microfluidic device, Angiogenesis, Revascularization, Fibrin hydrogel, Angiogenic response, Endothelial

## Abstract

Fibroblasts play indispensable roles in orchestrating revascularization and angiogenesis of endothelial cells. However, it remains elusive the specific conditions under which the influence of fibroblasts becomes important in modulating angiogenic formation. Here, we extend a previously established microfluidic co-culture system to enable precise manipulation of the density of human lung fibroblasts (HLF) and their distance to human umbilical vein endothelial cells (HUVECs) in a wide range within fibrin hydrogels. Using live cell imaging and image analysis, we quantify the effects of fibroblast density, HLF-HUVEC cell-cell distance, and culturing time on cell migration, morphology, microvasculature, and endothelial sprouting. We find that, for fibroblasts to have a significant positive impact in the angiogenesis of endothelial cells, the fibroblast density should be no less than ~3×10^6^ cells/ml and the cell-cell distance should be no greater than ~2 mm. The experimental findings can be captured by a minimal model accounting for the concentration profile of pro-angiogenic factors secreted by fibroblasts. Our results provide insights into the remodeling of vasculature and the guidance for engineering vascularized tissue mimics.

## Introduction

Angiogenesis, the formation of new blood vessels from existing vasculature, is essential for various biological processes including tissue regeneration^1,2^, wound healing^2,3^, and cancer progression^4,5^. As the primary cellular component of blood vessels, endothelial cells line the inner surface of vessels to support tissue growth and repair. While endothelial cells are the primary drivers of vascular formation, their behavior is profoundly influenced by surrounding stromal cells, particularly fibroblasts, which modulate angiogenesis through biochemical signaling and extracellular matrix remodeling^6–9^. Coordinated interactions among endothelial cells, mural cells, and stromal tissues govern sprouting, migration, lumen formation, and vessel stabilization during vascular development^3^. In parallel, ECM remodeling modulates angiogenesis by altering biochemical composition and mechanical properties, including growth factor sequestration, matrix stiffness, and bioactive matrix fragments that influence endothelial proliferation and sprouting^10^. In vitro co-culture models further demonstrate the pro-angiogenic role of fibroblasts. For instance, by pre-mixing human umbilical vein endothelial cells (HUVECs) and human lung fibroblasts (HLF) and culture them within a fibrin gel, an early co-culture system showed that fibroblasts could enhance endothelial sprouting and the formation of capillary networks via paracrine signaling mechanisms^11^. Moreover, fibroblasts can secrete matrix proteins to stiffen fibrin gel that correlates with enhanced lumen formation^12^. However, these co-culture systems have limited control over the proximity of fibroblasts to endothelial cells.

Unlike conventional co-culture systems, microfluidic platforms allow patterning fibroblasts and endothelial cells at controlled distances within hydrogels^13–18^. This lab-on-chip platform enables engineering perfusable three-dimensional (3D) microvascular networks and tumor vasculatures based on the spatially controlled co-culture of endothelial cells with other cell types in fibrin gels^13^. However, in this classic microfluidic co-culture system, two fibroblast channels symmetrically distribute on the two sides of a HUVECs channel^13^. This double-side geometric configuration inevitably creates a non-monotonic distribution in fibroblasts secreted pro-angiogenic factors with concentration peaks at location with equal distance from the two fibroblast channels. This may complicate the understanding of effects of HLF-HUVEC cell-cell distance on the angiogenic response of HUVECs, which is known to be sensitive to the concentration and distribution of pro-angiogenic factors. Additionally, only a limited range of fibroblast density (5 to 8 ×10^6^ cells/ml) and HLF-HUVEC cell-cell distance (500 to 1000 μm) have been explored. Consequently, quantitative data linking specific fibroblast densities and spatial configurations to angiogenic efficiency remains limited.

**Figure 1.**
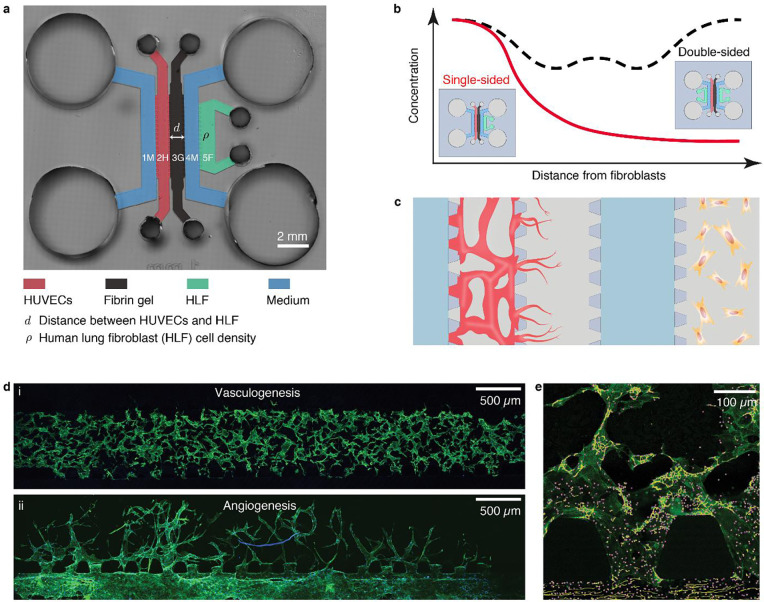
A microfluidic device for studying the formation of microvascular networks regulated by the proximity and cell density of fibroblasts. **(a)** A tile-scan image of the microfluidic device. The cell-cell distance *d* and fibroblast density *ρ* can be independently controlled to study their roles in the formation of the microvascular network and sprouts. From left to right channels: 1M for culture medium, 2H for fibrin hydrogel only, 3F for fibroblasts, 4M for culture medium, and 5F for fibroblasts. **(b)** A schematic that shows the difference in the concentration profile of growth factors between the classical single-sided and our double-sided cell seeding configuration. **(c)** A schematic of the microfluidic device in **(a)** to illustrate the formation of microvascular network and sprouts from the endothelial cell channel. **(d)** Tile scan fluorescence confocal images of pre-vascularized network and angiogenetic sprouts in microfluidic channels. (i) Microvascular network within the endothelial channel, (ii) and angiogenic sprouting inside the Channel 3G after 48 hours, using a 1 mm-wide acellular channel and seeding density of 3×10^6^ cells/ml for HUVEC and 5×10^6^ cells/ml for HLF. **(e)** Fluorescent microbeads of 1 μm in diameter are introduced from Channel 1M and transport within the microvascular network. The trajectories of these microbeads, represented by yellow lines, illustrate their transport paths through the interconnected luminal spaces of the vascular structure.

Here, we extend the classic double-side microfluidic co-culture device to a single-side system (Fig. 1a) to achieve a monotonic gradient profile of fibroblasts secreted pro-angiogenic factors (Fig. 1b). We systematically vary the fibroblast density within a wide range from 1 to 10 ×10^6^ cells/ml and the HLF-HUVEC cell-cell distance from 1 to 10 mm. Using live cell imaging and image analysis, we quantify the dependence of cell migration, morphology, microvasculature, and endothelial sprouting on fibroblast density, cell-cell distance, and culturing time. We find that, for fibroblasts to have a significant positive impact in the angiogenesis of endothelial cells, the fibroblast density should be no less than ~3×10^6^ cells/ml and the cell-cell distance should be no greater than ~2 mm. Accounting for the concentration profile of pro-angiogenic factors secreted by fibroblasts, we develop a minimal model that successfully captures the dependence of sprout length on fibroblast density and cell-cell distance. Our results provide insights into the remodeling of vasculature and the guidance for engineering vascularized tissue mimics.

## Results and Discussion

### Design and fabrication of a microfluidic device for studying microvasculature

Fibroblasts secrete a variety of pro-angiogenic factors, such as vascular endothelial growth factor (VEGF), basic fibroblasts growth factor (bFGF), platelet-derived growth factor (PDGF), and stromal-derived factor 1 (SDF-1), which collectively stimulate endothelial cell proliferation, migration, and tube formation^19–22^. Thus, the formation of vasculature is determined by both the concentration and the gradient of pro-angiogenic factors sensed by HUVECs^23,24^. These two parameters are expected to be dependent on both (i) the distance between HUVECs and HLF and (ii) the density of HLF. To explore this, we design a microfluidic device that consists of five adjacent channels, which are consecutively loaded with cell culture medium (Channel 1M), HUVECs (Channel 2H), fibrin gel (Channel 3G), cell culture medium (Channel 4M), and HLF (Channel 5F), as illustrated from left to right in Fig. 1a. The dimension of the channels is fixed at 1 mm in width (except for fibrin gel Channel 3G) and 150 μm in height, large enough for monitoring the angiogenesis within a few days. Moreover, the medium reservoir has a diameter of 6 mm and a height of 1.5 mm (**Fig. S1**), sufficient for supplying nutrients for cell growth for at least 48 hours, by which the culture medium is changed. Note that our design is inspired by but different from the classic microfluidic device, which have two stromal fibroblast channels symmetrically distributed on the two sides of a Channel 2H^13^; this geometric configuration inevitably creates non-monotonic distribution in fibroblasts secreted pro-angiogenic factors with concentration peaks at location with equal distance from the two fibroblast channels, as illustrated by the dashed line in Fig. 1b. By contrast, our design uses a single fibroblast channel to achieve a gradient profile of fibroblasts secreted pro-angiogenic factors (red line in Fig. 1b). Moreover, the concentration of pro-angiogenic factors at the Channel 2H can be tuned by either the concentration of the HLF or the width of the acellular fibrin gel Channel 3G that separates the HUVECs and the HLF.

In the microfluidic device, instead of using relatively thick cover slides, we use a thin cover slip as the substrate. This allows using lens with relatively high magnification and short working distance to obtain high-resolution images of cell morphology. Additionally, we pre-inject the microfluidic channels with carbon dioxide (CO_2_), which can dissolve into water; this avoids trapped air bubbles when loading the fibrin gel and cells.

Besides the channels for cell culture medium, all channels are loaded with fibrin gel, which is formed by thrombin-catalyzed conversion of fibrinogen to fibrin, a process mimicking that in blood clotting. For the channel containing cells, the cells are pre-mixed with the fibrin gel at a prescribed cell density. The concentration of the fibrin gel is fixed 2.5 mg/ml, corresponding to 7.4 μM, at which the diameter of the fibrin fiber is about *d* ≈ 1 *μm*^25^. Since fibrin is a semiflexible biopolymer, the mesh size of the fibrin gel is determined by the fibrin volume fraction *ϕ* and the fiber diameter $d:\xi \approx d\phi^{-1/2}\approx 20\;\mu m$^26^. This pore size is large enough to ensure sufficient transport of nutrients and oxygen for cell growth and migration. Moreover, the concentration of thrombin within the fibrinogen is 1 U/ml (~ 200 nM), resulting in a crosslinked fibrin gel with a shear modulus 200 Pa, as shown by the small amplitude oscillatory shear measurements in **Fig. S2**. This stiffness is high enough to mechanically support cell infiltration and the formation of capillary-like structure^27,28^ (Fig. 1c). Additionally, the degradation rate of the fibrin gel of this concentration is fast enough to accommodate matrix remodeling during tissue formation^29^. Indeed, our microfluidic setup allows for vasculogenic development, as exemplified by a tile scan fluorescence image of the whole device, which shows a microvascular network (μVN) (panel i in Fig. 1d) formed by HUVECs expanding into the adjacent channel filled with fibrin gel only (panel ii in Fig. 1d). We note that fibrin gels exhibit batch-to-batch variability and possess inherent translational and mechanistic limitations. The specific gel composition and mechanical properties used here are selected to provide a reproducible and pro-angiogenic environment for controlled spatial interrogation. Extension of this framework to alternative natural or synthetic matrices will be important for future translational studies.

To further validate the functionality of the engineered microvascular networks, we assessed their perfusability using 1 μm fluorescent microbeads under hydrostatic pressure. Specifically, we load a suspension of microbeads into the microfluidic device from one of the two medium reservoirs for HUVECs. Within a few seconds, the microbeads enter the vascular network through the medium Channel 1M and transport through the interconnected vessel structures, as shown by Fig. 1e and **Movie S1**. Moreover, tracking the bead trajectories confirms that the microbeads are confined within the vessel lumen, as shown by the yellow lines in Fig. 1e. These results demonstrate that our microfluidic co-culture system allows for engineering perfusable microvasculature in vitro.

### Effects of cell-cell distance on microvasculature

We define cell-cell distance as the separation distance between HUVEC and HFL cell populations. To explore the effects of cell-cell distance on the formation of microvasculature, we fix the concentrations of HUVECs and fibroblasts, respectively, at 3 and 5 ×10^6^ cells per ml, comparable to those in existing studies^30^. We vary the width of fibrin gel Channel 3G, or the distance between the HUVEC and fibroblast channels, in a wide range from 1 to 10 mm, which is greater than the typical distance 1 mm explored in literature^30^. The cells are fluorescently labeled by cell tracker for live cell imaging without impairing their biological activity^31^. Since cell trackers typically allow for live cell imaging for up to 3 days, following initial staining, the cells are re-stained to extend the 5-day experiment window.

**Figure 2.**
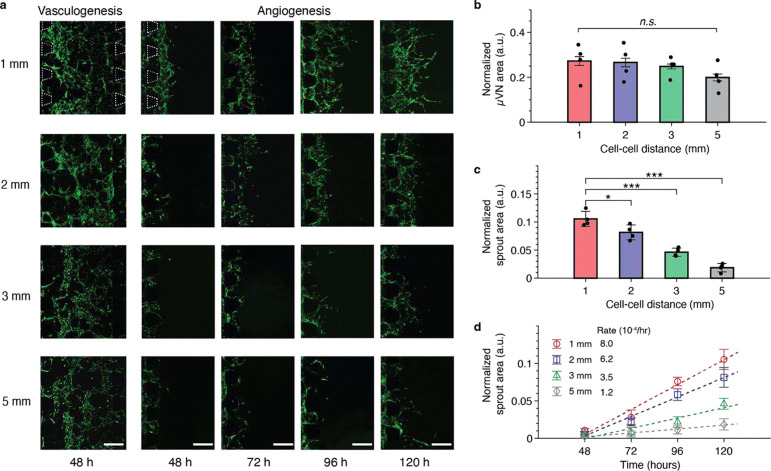
Vasculogenesis and angiogenesis at various cell-cell distances. The concentrations of HUVECs and fibroblasts are fixed at 3×10^6^ cells/ml and 5×10^6^ cells/ml, respectively. By contrast, the distance between the HUVEC and fibroblast channels varies from 1 mm to 5 mm. **(a)** Representative fluorescence confocal images of the vasculogenesis and angiogenesis channels over 120 hours. **(b)** Normalized microvascular network area in the vasculogenic channel at 48 hours. **(c)** Normalized area of sprouts, defined as area of the sprouts divided by the total area of the sprouting region, at 120 hours. **(d)** Dependence of normalized area of sprouts on culturing time for various cell-cell distances. *p<0.05; **p<0.01; ***p<0.001; *n.s.*, not significant; *n*=4.

Within the Channel 2H, after being cultured for 48 hours, the HUVECs start form microvascular network regardless of the distance from the fibroblasts, as shown by the representative fluorescence images on the left column of Fig. 2a. We use total sprout area as the primary angiogenic metric because it provides a robust, non-directional measure of global endothelial invasion driven by fibroblast-secreted growth factor gradients. This metric integrates collective cell migration, proliferation, and matrix remodeling into a single quantitative descriptor, minimizing variability arising from local morphological heterogeneity. Quantitatively, the normalized area of microvasculature, defined as the area of microvascular network normalized by the total area of the HUEVC channel, is about 25% and exhibits no significant difference across various cell-cell distance (Fig. 2b). This behavior is consistent with existing understanding that fibrin gel provides a favorable environment for vasculogenesis of HUVECs^28^. By contrast, the ability of HUVECs to invade the adjacent empty fibrin gel Channel 3G strongly depends on the cell-cell distance. For instance, at 48 hours, only for the case with 1 mm cell-cell distance, HUVECs exhibit some extent of angiogenesis behavior by migrating into Channel 3G to form a network (second column to the left in Fig. 2a). The difference in angiogenesis becomes dramatic after 120 hours, at which the normalized sprouting area, defined as the area of the sprouts divided by the total area of the sprouting region, decreases significantly from 10% to 2% as the cell-cell distance increases from 1 to 5 mm, as visualized by the right column in Fig 2a and quantified in Fig. 2c.

To further explore the angiogenic sprouting in response to the cell-cell distance, we quantify the dependence of the normalized sprouting area on culturing time for various cell-cell distances (Fig. 2d). Interestingly, the normalized sprouting area increases nearly linearly with culturing time. As the cell-cell distance increases by five times from 1 to 5 mm, the normalized sprouting rate, defined as the normalized sprouting area divided by culturing time, decreases by nearly 7 times from 8.0×10^−4^ to 1.2×10^−4^ per hour. Consistent with this observation, the spout diameter increases with the decrease in cell-cell distance (**Fig. S4a**). However, further increasing the cell-cell distance to 10 mm does not decrease the sprouting area (**Fig. S3**). These phenomena are likely because of the concentration of angiogenic factors secreted by fibroblasts decreases with the increase of cell-cell distance, and that at 10 mm distance, the concentration becomes too low to induce cell response. These results justify that the largest separation condition serves as an effective no fibroblast control.

### Effects of fibroblast density on microvasculature

To explore the effects of fibroblast density on vasculogenesis and angiogenesis, we fix the cell-cell distance at 1 mm and the concentration of HUVECs at 3×10^6^ cells/ml, while changing the concentration of HLF from 0 to 10 ×10^6^ cells/ml. Higher HLF concentrations (>10×10^6^ cells/ml) markedly increases the viscosity of cell suspension, posing challenges in loading the cells into the microfluidic channels. Nevertheless, the range of cell density explored here is wider than 5 to 8×10^6^ cells/ml in previous studies^13,30,32^.

**Figure 3.**
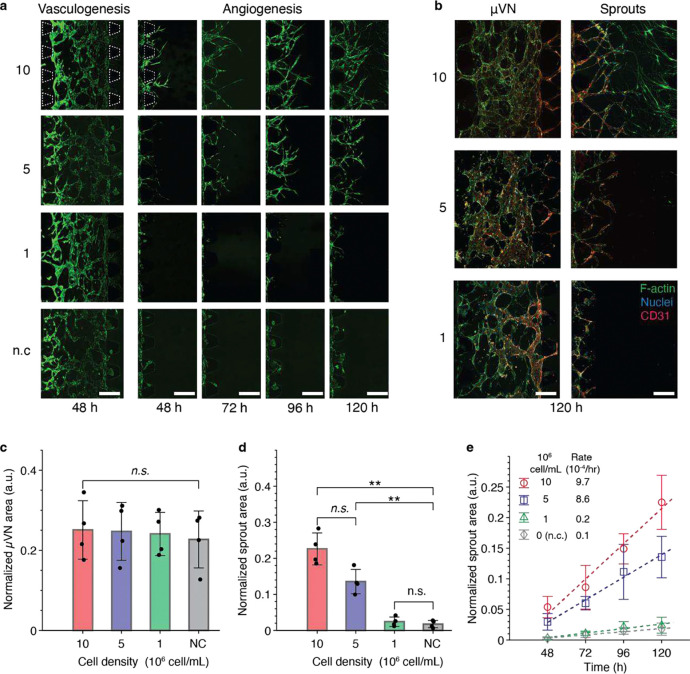
Effects of fibroblast density on microvasculature. The distance between the HUVEC and fibroblast channels is fixed at 1 mm, and the seeding cell density of HUVECs is maintained at 3×10^6^ cells ml^−1^. By contrast, the density of fibroblasts is varied from 0 (negative control, n.c.) to 10×10^6^ cells ml^−1^. **(a)** Representative fluorescence images for various fibroblast densities over 120 hours. **(b)** Immunofluorescence images of angiogenic sprouts (HUVECs, red) and the proliferation of fibroblasts (green) within the fibrin gel Channel 3G. **(c)** The normalized microvascular network area at 48 hours. **(d)** The normalized sprouting area at 48 hours. **(e)** Dependence of the normalized area of spouts on culturing time. Dashed lines: The sprouting rate for various cell densities. *p<0.05; **p<0.01; ***p<0.001; *n.s.*, not significant; *n*=4.

Interestingly, vasculogenesis of HUVECs within the fibrin gel is nearly independent of the HLF concentration. By contrast, the process of angiogenesis strongly depends on HLF concentration. For instance, at 48 hours, the normalized area of microvasculature is nearly constant (24±6)% regardless of the HLF concentration (fluorescence confocal images on the left of Fig. 3a and data in Fig. 3c). By contrast, at 48 hours, the normalized sprouting area increases by nearly five times from (2±1)% to (23±4)% as the HLF concentration increases from 1 to 10 ×10^6^ cells/ml (second column to the left in Fig. 3a and data in Fig. 3d). Yet, there is no significant difference in sprouting between the 1 ×10^6^ cells/ml and the negative control without HLF.

The effects of HLF cell density on angiogenesis are further confirmed by the sprouting rate. Over the 5-day culturing period, for the HLF cell densities explored, the normalized sprouting area increases linearly with culturing time (Fig. 3e). As the concentration of HLF cells decreases by nearly two times from 10 to 5 ×10^6^ cells/ml, the normalized sprouting rate decreases slightly from 9.7×10^−4^/hr to 8.6×10^−4^/hr (red and blue dashed lines in Fig. 3e). For HLF density of 5 ×10^6^ cells/ml, the sprouting rate (8.6×10^−4^/hr) is consistent with independent measurements (8.0×10^−4^/hr), as shown by the red circles in Fig. 2d. Further reducing the cell density to 1×10^6^ cells/ml leads to a sprouting rate of 0.2×10^−4^/hr, this sprouting rate has no significant difference compared to the control group without HLF. Similar trend is observed for the dependence of sprout diameter on cell density (**Fig. S4b**). These observations are consistent with the understanding that, below a threshold cell density, the concentration of signaling molecules secreted by HLF is insufficient for cell-cell commutations that rely on concentration-sensitive signaling pathways^33,34^. Taken together, our results unambiguously show that the threshold density of HLF to promote angiogenesis of HUVECs is approximately 1×10^6^ cells/ml.

Remarkably, for the HLF with the highest density of 10×10^6^ cells/ml, at relatively long culture time of Day 5, we observe a nonuniform cell density within the fibrin gel Channel 3G. Compared to that in the middle of Channel 3G, the cell density is relatively high with more cells located the left and right edges of the channel, which are, respectively, adjacent to the HUVECs Channel 2H and the culture medium Channel 4M. To further explore this behavior, we stain the F-actin and nuclei of all cells using phalloidin and Hoechst 33342, respectively. Moreover, we identify endothelial cells using a red fluorescent molecule tagged mouse monoclonal antibody against CD31, a transmembrane glycoprotein expressed on endothelial cells (Fig. 3b). This dual immunostaining reveals that the endothelial cells are mainly located at the side adjacent to the HUVECs Channel 2H. However, the cells adjacent to the culture medium Channel 4M are fibroblasts. These results suggest that the fibroblasts are not confined within their own channel at relatively long culture times. Instead, they migrate across the culture medium Channel 4M of 1 mm in width, which is nearly 50 times of the cell size, and enter the fibrin gel Channel 3G adjacent to Channel 2H. This behavior may arise from matrix deposition or surface conditioning that enhances adhesion along PDMS surfaces. Nevertheless, the invasion of fibroblasts likely enhances the concentration of growth factors at the sprouting sites^35,36^, correlating closely with the notably high angiogenic sprouting rate (9.7×10^−4^/hr) (Fig. 3e). However, reducing fibroblast density below 5×10^6^ cells/ml diminishes cell invasion that is associated with substantially decreased the sprouting rate (Fig. 3e). These results show that a threshold cell density is necessary to sustain sufficient pro-angiogenic signaling, highlighting the synergistic effects of fibroblasts invasion, local biochemical signals, and effective angiogenesis.

### Modeling sprout length induced by fibroblasts

We develop a phenomenological model to describe the effects of fibroblast density and cell-cell distance on sprout length. To construct the phenomenological model, we quantify sprout length rather than sprout area at later stages of angiogenesis (Day 5). At this time point, endothelial sprouts exhibit defined, elongated morphology, and sprout length measured perpendicular to the cell channels directly reflects directional extension toward fibroblast-derived signals. In contrast, sprout area is more appropriate for early-stage analysis (Day 1–3), where cell invasion is collective and individual sprouts are not yet well defined..

The sprout length is proportional to migration speed of endothelial cells, which is determined by the concentration of pro-angiogenic signaling factors secreted by the fibroblasts. For a fixed cell-cell distance, the higher the density of fibroblasts, the higher the concentration of pro-angiogenic factors. However, for living cells, the concentration of secreted molecules is not linearly proportional to the cell density. Instead, the dependence of the concentration of secreted molecules typically consists of three phases: slow initial increase, rapid exponential increase, and eventually saturation due to limited resources for cell growth and activity. This behavior is often described by sigmoid function^37^:

(1) $$\ell (\rho ,d)=\frac{\ell_{d}}{1\;+\;exp\left[-\frac{\rho \;-\;\rho_{0}}{\rho_{c}}\right]}$$

Here, ℓ is the sprout length, ρ is the density of fibroblasts, $\rho_{0}$ is the crossover cell density above which the HLF start to promote the angiogenesis, $\rho_{c}$ is the characteristic cell density of the exponential growth, and $\ell_{d}$ describes the characteristic sprout length at certain cell-cell distance d.

For a fixed fibroblast density, as the cell-cell distance d increases, the concentration of secreted pro-angiogenic factors decreases, and so does the sprout length. However, the sprout length remains finite even without the presence of fibroblasts, as HUVEC cells themselves can proliferate and migrate within fibrin gel, resulting in vasculogenesis and then angiogenesis^27,38^. Moreover, as the cell-cell distance is above a characteristic value, the impact of fibroblasts on the angiogenesis of endothelial cells is expected to diminish, which often follows an exponential decay^39,40^. Thus, we use an offset exponential function to describe the impact of cell-cell distance on sprout length:

(2) $$\ell_{d}=\ell_{0}+\ell_{\max}exp\left(-\frac{d}{d_{c}}\right)$$

Here, $\ell_{0}$ is the sprout length intrinsic to the proliferation and migration of endothelial cells themselves, $\ell_{\text{max}\;}$ is the maximum sprout length when the two cell types are in contact, and $d_{c}$ is the characteristic cell-cell distance below which fibroblasts strongly impact the behavior of endothelial cells. Note that we ignore the contribution of the medium channel (1 mm in width) to the cell-cell distance d, as the diffusion coefficient of pro-angiogenic factors such as VEGF in culture medium (~10^−9^ m^2^/s) is about two orders of magnitude higher than that in fibrin gel (~10^−11^ m^2^/s)^41,42^. Thus, it is reasonable to assume that the concentration of fibroblasts secreted pro-angiogenic factors is uniform within the fibroblast and medium channels, and that the cell-cell distance is largely determined by the width of the fibrin gel Channel 3G.

**Figure 4.**
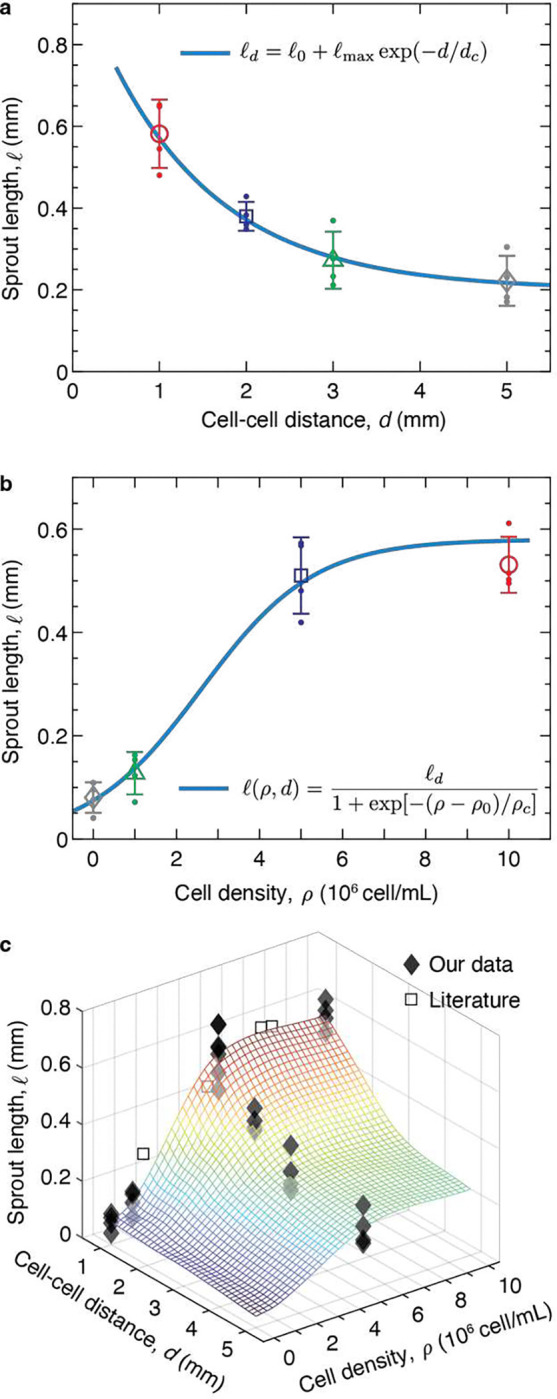
Modeling of sprout length as a function of cell-cell distance and fibroblasts seeding density. **(a)** Sprout length, <mi>, as a function of cell-cell distance, *d*. Symbols: data points represent mean values with error bars indicating standard deviations. Solid line: Weighted fit using sigmoid function [eq. (1)]: $\ell_{d}=0.54\pm 0.06\;mm,\rho_{0}=2.1\pm 0.8\times 106$ mm, *ρ*_0_ = 2.1 ± 0.8 ×106 cells/ml, and *ρ*_*c*_ = 1.1 ± 0.5 ×106 cells/ml. **(b)** Sprout length as a function of cell density, *ρ*. Solid line: Weighted fit using eq. (2) with fitting parameters $\ell_{\text{max}\;}=0.8\pm 0.3\;mm$ and *d*_*c*_ = 1.3 ± 0.7 mm. $\ell_{0}=0.2\;mm$ is the sprout length intrinsic to the proliferation and migration of endothelial cells themselves. **(c)** Diagram-of-state for the dependence of the sprout length on cell density and cell-cell distance. Filled symbols: our data; empty symbols: data from literature with cell-cell distance of 0.7 mm.

We determine the coefficients of the models by fitting them to the two sets of experimental data. Our experiments show that for a fixed fibroblast density *ρ* = 5 × 10^6^ cells/ml, the sprout length is nearly constant 0.2 mm at cell-cell distance *d* ≥ 5 mm. This gives $\ell_{0}=0.2\;mm$ mm and reduces eq. (2) to a two-parameter model. The model describes the experimental data remarkably well (Fig. 4a) with fitting parameters of $\ell_{\text{max}}=0.8\pm 0.3\;mm$ and *d*_*c*_ = 1.3 ± 0.7 mm. These results suggest that the cell-cell distance should be no more than 2 mm for fibroblasts to have a significant positive impact on angiogenesis of endothelial cells.

Similarly, our minimal model [eq. (1)] describes well the experimental data on the dependence of sprout length ℓ on fibroblast density ρ for a fixed cell-cell distance (Fig. 3b). The best fit gives $\ell_{d}=0.54\pm 0.06\;mm,\rho_{0}=2.1\pm 0.8\times 10^{6}$ cells/ml, and *ρ*_*c*_ = 1.1 ± 0.5 ×10^6^ cells/ml. These results indicate that for a fixed cell-cell distance *d* = 1 mm, the maximum sprout length that can be achieved by increasing fibroblast density is $\ell_{d}=0.54\pm 0.06\;mm$. Moreover, if the fibroblasts concentration is above $\rho >\rho_{c}+\rho_{0}\approx 3.2\pm 1.3\times 10^{6}$ cells/ml, the effects of fibroblast density saturate and thus the spout length will be maximized. Consistent with this understanding, eq. (2) predicts that for *ρ* = 5 × 10^6^ cells/ml and *d* = 1 mm, the sprout length will be $\ell_{max}\approx 0.58\;mm$, comparable to the maximum sprout length predicted by eq. (1). Note that our phenomenological models are based on limited conditions explored in this study, and extension to additional endothelial–stromal combinations would further test the model. Nevertheless, the prediction from our minimal model successfully captures the behavior reported in literature that measured the sprout length for cell-cell distance of 0.7 mm (empty squares, Fig. 4c). Taking together, these results suggest that, for fibroblasts to have significant positive impact in the angiogenesis of endothelial cells, the fibroblast density should be no less than ~3×10^6^ cells/ml and the HLF-HUVEC cell-cell distance should be no greater than ~2 mm, [*ρ* ≥ ~3 × 10^6^ cells/ml, *d* ≤ ~2 mm], as shown the plateau region in Fig. 4c.

### Conclusion and Outlook

In summary, we have developed microfluidic co-culture system and exploited it to systematically investigate the effects fibroblast densities and spatial configurations to angiogenic efficiency of endothelial cells. We vary the fibroblast density from 1 to 10 ×10^6^ cells/ml and the HLF-HUVEC cell-cell distance from 1 to 10 mm; these ranges are much wider than those explored in literature. Using live cell imaging and image analysis, we quantify the dependence of cell migration, morphology, microvasculature, and endothelial sprouting on fibroblast density, cell-cell distance, and culturing time. For fibroblasts to have a significant positive impact in the angiogenesis of endothelial cells, the fibroblast density should be no less than ~3×10^6^ cells/ml and the cell-cell distance should be no greater than ~2 mm.

Like the classic microfluidic co-culture system^13^, our system enables engineering perfusable vasculature in vitro. The difference is that our co-culture system allows for generating a monotonic gradient profile of pro-angiogenic factors secreted by fibroblasts. This simplifies the understanding of the effects of fibroblast density and spatial proximity in angiogenic response of endothelial cells. Accounting for the concentration profile of pro-angiogenic factors secreted by fibroblasts, we have developed a phenomenological model that successfully captures the dependence of sprout length on fibroblast density and cell-cell distance. Note that our model does not distinguish the roles of specific pro-angiogenic factors secreted by fibroblasts as well as the physical properties of fibrin gels, which may impact the angiogenic outcomes^39,43–45^, and we did not systematically evaluate donor-to-donor variability in HUVECs or HLFs. Although different cell lots may alter absolute sprouting magnitude, the spatial scaling trends and threshold behaviors identified here arise from defined geometric constraints and are therefore expected to remain robust across sources. By establishing quantitative relationships between fibroblast distribution, density, and angiogenic outcomes, our results provide actionable guidelines for engineering functional vasculature. This becomes particularly useful with the advent of 3D bioprinting technologies, which offer exquisite control over the cell location in 3D space^46–48^.

Although the HUVEC/HLF co-culture model is widely used, it has inherent limitations^49^. Endothelial cells exhibit significant tissue-specific heterogeneity, and stromal populations such as fibroblasts or mesenchymal stem cells (MSCs) differ in their paracrine and matrix remodeling profiles. The relationships identified here are therefore not intended to represent universal biological constants. Rather, they define a spatial–density framework for interpreting angiogenic responses. Because our microfluidic platform is cell-agnostic, the same experimental parameters can be directly applied to more physiologically relevant endothelial sources, such as human induced pluripotent stem cell (iPSC)–derived endothelial cells (ECs)^50–52^, and alternative stromal populations. This approach provides a foundation for systematically evaluating how different EC–stromal combinations interpret spatial and density cues, advancing the development of more translatable in vitro angiogenesis models.

## Materials and Methods

### 1. Fabrication of microfluidic devices

We use soft lithography^53,54^ to fabricate microfluidic devices in poly(dimethyl siloxane) (PDMS). The fabrication process involves five steps: (i) mask design using computer-aided design (CAD) software, (ii) mask printing (Artnet Pro Inc), (iii) fabrication of the SU8-on-Si wafer masters (SU8-2150, Microchem, USA), (iv) curing of the PDMS (Sylgard 184, Dow Corning) using the master as a mold, and (v) bonding of the PDMS slabs to a glass substrate.

### 2. Cell culture

Primary human umbilical vein endothelial cells (HUVECs PCS-100-010, ATCC) are cultured in endothelial growth medium (Kit-VEGF PCS-100-041, ATCC). Normal primary human lung fibroblasts (HLF PCS-201-013, ATCC) are cultured in fibroblasts growth medium (Kit-Low serum PCS-201-041, ATCC). HUVECs and HLF are ready for the experiments after passages 3 to 5 and 5 to 7, respectively. All cells are maintained in a humidified incubator at 37 °C and 5% CO_2_.

### 3. Angiogenesis assay

We prepare a solution of 10 mg/ml fibrinogen (F8630, Sigma-Aldrich) and 0.45 U/ml aprotinin (A1153, Sigma-Aldrich) in Dulbecco’s phosphate-buffered saline (DPBS, Hyclone). In parallel, HUVECs are collected and suspended in cell culture medium at a concentration of 6.67×10^6^ cells/ml, and fibroblasts are prepared at concentrations ranging from 1.33×10^6^ to 13.3×10^6^ cells/ml. We mix the fibrinogen solution with the cell suspension at 1:3 volume ratio, resulting in final concentrations of 2.5 mg/ml fibrinogen, 3×10^6^ cells/ml^−^ HUVECs, and 1×10^6^ to 10×10^6^ cells/ml fibroblasts.

Before loading hydrogels, we pre-fill the channels with CO_2_, which can dissolve into water to avoid the formation of air bubbles trapped between adjacent channels and within the micro post structures. To load fibroblasts into the microfluidic device, we add thrombin (T4648, 50 U/ml, Sigma-Aldrich) to the fibroblast–fibrinogen mixture at 50:1 volume ratio and immediately inject the mixture into Channel 5F. We wait for 3 minutes to allow for fibrin cross-linking and then load a fibrinogen solution (2.5 mg ml^−1^ in PBS) mixed with thrombin into the fibrin gel Channel 3G, followed by a 3-minute clotting period to form the acellular fibrin gel. We then fill the Channel 2H with the HUVEC–fibrin gel mixture using the same method as that for Channel 5F. After waiting for 3 minutes, we fill the upper reservoirs of each device with endothelial cell growth kit-VEGF culture medium (PCS-100-04, ATCC). The lower reservoirs are gently aspirated to ensure proper filling of Channel 1M and 4M, and all reservoirs are then filled up and leveled with Kit-VEGF medium to establish static pressure between the two medium channels. We change the cell culture medium 24 hours after cell seeding and refresh it every 48 hours thereafter.

### 4. Live cell imaging and immunostaining

For live cell imaging, we use cell tracker to stain the cells. Specifically, CellTracker^™^ Green CMFDA dye (Invitrogen, MA) is diluted in pre-warmed, serum-free basal medium to a final concentration of 10 μM. After removing the culture medium from Channels 1M and 4M and reservoirs, the channels are rinsed with phosphate buffered saline (PBS, Hyclone) to eliminate residual medium. The CellTracker^™^ solution is then added to the channels, and the device is incubated for 40 minutes at 37 °C. Following incubation, the staining solution is removed, and the channels are washed three times with PBS to remove residual dyes. Fresh, pre-warmed culture medium containing serum is added, and the cells are incubated for an additional 30 minutes to allow internalization of fluorescent dye. Fluorescence imaging is performed using a fluorescence confocal microscope (Leica, SP8) with filter settings of excitation/emission: 492/517 nm. To allow for live-cell imaging over 5 days, the sample is re-stained on Day 3 using the same procedure.

For immunofluorescence staining, we wash the cultured samples once with PBS and fix the cells in 4% (w/v) paraformaldehyde in PBS for 15 mins, then permeabilize the samples using a 0.15% Triton X-100 (Sigma) in PBS solution for 15 min. After blocking with 3% bovine serum albumin (BSA, Sigma) in PBS for 1 hour, we incubate the samples overnight at 4 °C with antibody against CD31 (Alexa Fluor^®^ 647, clone WM59). To stain F-actin and DNA, we add Alexa Fluor^®^ 488-Phalloidin (66 nM) and Hoechst 33342 (1:1000) and incubate for 1 hour at room temperature. The samples are washed three times and stored in PBS before imaging.

### 5. Perfusability of microvascular network

To assess the perfusability of microvascular networks, after 4 days of vasculogenic and angiogenic vessel formation, we stain the sample using Alexa Fluor^®^ 488-Phalloidin to visualize endothelial structures. Then, we suspend 1 μm fluorescent polystyrene beads (F13080, Invitrogen) in PBS and introduce the suspension into the microfluidic device from one of the two medium reservoirs for HUVECs. Before doing so, the residual fluid from two medium reservoirs for HUVECs is aspirated. Note that the bead suspension is added to one reservoir only; this generates a concentration gradient that results in unidirectional flow of beads through the microvascular network. Microbead trajectories are analyzed using the TrackMate plugin in ImageJ. Individual bead paths are tracked and rendered as yellow lines overlaid on confocal images. Successful perfusion is defined by the presence of continuous bead trajectories confined within the luminal structures of the microvascular network, confirming the formation of potent, interconnected endothelial channels capable of supporting fluid transport.

### 6. Image and statistical analysis

Angiogenic sprout areas and lengths are quantified using ImageJ. Fluorescent images of HUVECs are acquired every 24 hours after seeding, and each image is processed for background subtraction and thresholding to clearly define sprout boundaries. To ensure consistency, identical thresholding and measurement parameters are applied across all images in each experimental condition. Sprouts from four randomly selected regions of interest (ROIs) are analyzed for each condition. Measurements are averaged for each experimental group to ensure accurate quantification of angiogenesis.

For each experimental condition (e.g., specified gel separation distance or fibroblast density), we fabricate and prepare four microfluidic devices in parallel under identical configurations (*n* = 4). We seed and culture all replicates for a given condition on the same day to minimize variability associated with timing and handling. Across the full study, thirty-two devices successfully support angiogenic growth and are included in the analysis. An additional five to six devices fail during initial cell seeding, most commonly due to unintended leakage of the cell–gel suspension into adjacent channel regions, and we exclude these devices prior to data collection.

Statistical analysis is performed using one-way analysis of variance (ANOVA), followed by Tukey’s Honest Significant Difference (HSD) test to assess the significance of differences across all sprouting conditions for each group of study. Data are collected from multiple independent experiments using parallel microfluidic devices and are reported as mean ± standard deviation. All statistical calculations are conducted using SPSS (Microsoft, WA).

## Supplementary Material

Supplementary Information

Configuration and dimension of microfluidic device, mechanical properties of fibrin gel, data on angiogenesis, and supplementary videos.

## Data Availability

All data are available in the manuscript or supplementary information.
